# Lipid Droplet and Peroxisome Biogenesis: Do They Go Hand-in-Hand?

**DOI:** 10.3389/fcell.2019.00092

**Published:** 2019-05-31

**Authors:** Amit S. Joshi, Sarah Cohen

**Affiliations:** Department of Cell Biology and Physiology, The University of North Carolina at Chapel Hill, Chapel Hill, NC, United States

**Keywords:** lipid droplet, peroxisome, organelle biogenesis, membrane trafficking, lipid metabolism

## Abstract

All eukaryotic cells contain membrane bound structures called organelles. Each organelle has specific composition and function. Some of the organelles are generated *de novo* in a cell. The endoplasmic reticulum (ER) is a major contributor of proteins and membranes for most of the organelles. In this mini review, we discuss *de novo* biogenesis of two such organelles, peroxisomes and lipid droplets (LDs), that are formed in the ER membrane. LDs and peroxisomes are highly conserved ubiquitously present membrane-bound organelles. Both these organelles play vital roles in lipid metabolism and human health. Here, we discuss the current understanding of *de novo* biogenesis of LDs and peroxisomes, recent advances on how biogenesis of both the organelles might be linked, physical interaction between LDs and peroxisomes and other organelles, and their physiological importance.

## Introduction

Peroxisomes and LDs play important roles in cellular lipid metabolism. These organelles are major metabolic hubs in eukaryotic cells. Both organelles play roles in preventing cell toxicity, albeit in dissimilar ways (Kohlwein et al., 2013). Peroxisomes are sites for beta-oxidation of fatty acids in all eukaryotic cells (Mannaerts and Van Veldhoven, 1996). In yeast and plants, the entire pathway occurs on peroxisomes, whereas in animals it occurs in peroxisomes, and mitochondria (Kunau and Hartig, 1992). Other than oxidation of fatty acids, peroxisomes are essential for detoxification of hydrogen peroxide (Walker et al., 2017). In addition, peroxisomes are sites for synthesis of D-amino acids, plasmalogens, and certain precursors of cholesterol. Understandably, defects in peroxisome function lead to several metabolic disorders (Argyriou et al., 2017). These disorders are caused due to mutations in genes encoding peroxisomal biogenesis proteins (PEX) essential for peroxisome function (Gould and Valle, 2000). Taken as a group, peroxisomal disorders occur in 1 in 5000 individuals (Waterham et al., 2016). Some of the commonly known disorders include X-linked adrenoleukodystrophy and peroxisomal biogenesis disorders (PBD) such as Zellweger Syndrome. For detailed discussion on PBD and other peroxisome related metabolic defects we refer other reviews to the readers (Delille et al., 2006; Wanders, 2014). While peroxisomes are sites of lipid degradation, LDs are organelles that prevent cellular toxicity by sequestering and storing free fatty acids in neutral lipids such as triglycerides (TG) and sterol esters (SE) (Cohen, 2018). Recent studies have elucidated new roles of LDs in protein degradation and protection from ER stress and mitochondrial oxidative stress (Olzmann and Carvalho, 2018). Aberrant LD biogenesis is a hallmark of severe disorders including diabetes, atherosclerosis, lipodystrophy, and neurodegeneration (Krahmer et al., 2013; Onal et al., 2017).

## Peroxisome Biogenesis

Peroxisomes are organelles enclosed in a single bilayer membrane. Peroxisomes were observed in early electron micrographs as distinct membrane bound organelles highly associated with the ER (Baudhuin et al., 1965; de Duve and Baudhuin, 1966; Tsukada et al., 1968; de Duve, 1969; Novikoff and Novikoff, 1972). It was later demonstrated that, similar to mitochondria and chloroplasts, peroxisomal matrix proteins are directly imported post-translationally from cytosolic ribosomes. Thus, peroxisomes were known as semi-autonomous organelles that follow the growth and division model where a new organelle is formed from the pre-existing one (Lazarow, 1983; Goldman and Blobel, 2006). Under normal physiological conditions, mature peroxisomes receive membrane lipids and proteins from the ER membrane, which contributes to peroxisome growth prior to division (Motley and Hettema, 2007).

**FIGURE 1:**
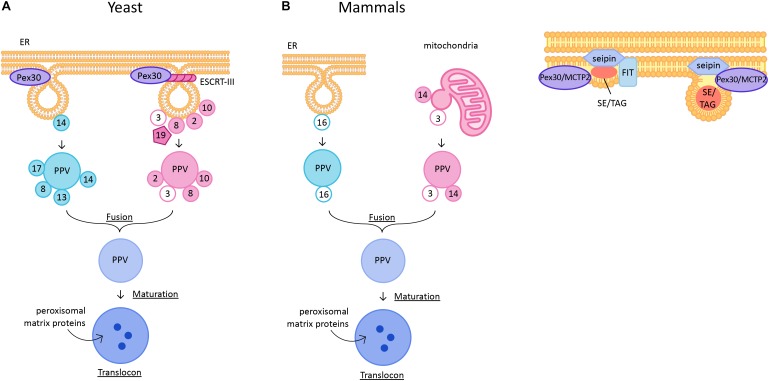
**(A)** *De novo* peroxisome biogenesis in yeast and mammalian cells. PPVs containing the docking complex proteins are generated independently of Pex3 and Pex19 at ER subdomains containing Pex30. PPVs containing ring complex proteins are generated in a Pex3- and Pex19-dependent manner. ESCRT-III complex is required for scission of PPVs from the ER membrane. The two pools of PPVs fuse to form an import competent mature peroxisome. In mammals, Pex16-containing PPVs from ER and Pex3 and Pex14-containing PPVs from mitochondrial outer membrane fuse to form a functional peroxisome. **(B)** Lipid droplet biogenesis. Neutral lipid synthesizing enzymes generate sterol esters (SE) and triglycerides (TG) to form a lens-like structure in the lipid bilayer. The ER regions are enriched with seipin, FIT proteins, and Pex30/MCTP2. As the lens grows the LD buds into the cytoplasm. In yeast, LDs remain attached to the ER subdomains containing Pex30/MCTP2 and seipin but no FIT proteins.

In yeast, growth and division is the major pathway of peroxisome biogenesis. However, peroxisomes can also originate by *de novo* biogenesis from the ER (Figure 1A; Hoepfner et al., 2005). This pathway was discovered when peroxisome biogenesis mutants (*pex*) devoid of functional peroxisomes were isolated using genetic screens in yeast (Liu et al., 1992; Van der Leij et al., 1992). These mutants exhibited ghost vesicles which were devoid of many matrix proteins. Depletion of some proteins such as Pex3, a peroxisomal membrane protein (PMP) required for localization and stability of other PMPs, Pex19, a chaperone, and receptor for PMPs, and Pex16, a PMP that can recruit Pex3, resulted in cells that were completely devoid of the ghost vesicles. This suggested that these proteins might play a role in the first steps of peroxisome vesicle formation (Hohfeld et al., 1991; Hettema et al., 2000; Tam et al., 2005; Kim et al., 2006; Fujiki et al., 2014; Erdmann et al., 2015). Interestingly, when the missing protein was re-introduced in these cells, mature peroxisomes reappeared, thus challenging the growth, and division model. The resulting *de novo* biogenesis model has two main aspects: formation of new pre-peroxisomal vesicles (PPVs) from a mother organelle followed by targeting of PMPs to these vesicles to form functional mature peroxisomes. The mother organelle is usually ER, as many PMPs are targeted to ER membrane in the absence of functional peroxisomes (Geuze, 2003; van der Zand et al., 2010; Agrawal and Subramani, 2013; Tabak et al., 2013; Kim and Hettema, 2015). However, in the absence of peroxisomes, many PMPs are mistargeted to mitochondria in mammalian cells (Kim and Hettema, 2015). The PMPs possibly leave the ER membrane in the newly formed PPVs (Agrawal et al., 2016; Joshi et al., 2016). Two independent *in vitro* studies reported an essential role for Pex19 in formation of nascent vesicles from the ER membrane. These studies demonstrated that PMPs such as Pex3, Pex15 (a tail-anchored PMP), and Pex11 (required for peroxisome proliferation), are targeted to the ER membrane and traffic to PPVs in a Pex19- and ATP-dependent manner (Agrawal et al., 2011; Lam et al., 2011). The targeting of PMPs to peroxisomes is independent of COPI and COPII proteins (South et al., 2000, 2002; Voorn-Brouwer et al., 2001).

In mammalian cells peroxisomes form through division of preexisting peroxisomes, but can also derive *de novo* under special conditions. Mammalian peroxisome biogenesis differs from yeast in that mitochondrial outer membrane can be a site for PPV formation (Figure 1A). Artificial targeting of Pex3 to mitochondrial outer membrane in Pex3-deficient cells resulted in *de novo* peroxisome biogenesis (Rucktäschel et al., 2010). Furthermore, a recent study demonstrated that there are two types of PPVs, one from ER that contains Pex16 and another from mitochondrial outer membrane that contains Pex3 and Pex14. These PPVs fuse to form a functional peroxisome (Sugiura et al., 2017). Existence of more than one type of PPV is controversial in yeast cells. Earlier reports showed that the importomer complex consisting of RING complex (Pex2, Pex10, and Pex12) and docking complex (Pex13, Pex14, and Pex17) sorted in distinct PPVs and fused to form a functional peroxisome (Figure 1A; Van Der Zand et al., 2012). The model for existence of two pools of PPVs is consistent with the report that sorting of RING complex proteins, but not the docking complex proteins, in the ER membrane is dependent on Pex3 (Agrawal et al., 2016). Interestingly, the vesicles consisting of docking complex proteins exist in yeast cells lacking Pex3 or Pex19. Therefore, neither Pex3 nor Pex19 is essential for docking complex-containing PPV formation (Knoops et al., 2014; Wróblewska et al., 2017). Thus, there are two pools of PPVs: Pex3- or Pex19-independent PPVs that contain docking complex proteins, and Pex3- and Pex19- dependent PPVs that contain RING complex proteins. The Pex1-Pex6 AAA ATPase proteins are required for fusion of two pools of PPVs (Figure 1A; Van Der Zand et al., 2012). In *Y. lipolytica*, the two pools of PPVs were reported to require cytosolic factors, ATP hydrolysis, and functional ATPases (Pex1 and Pex6) for fusion (Titorenko et al., 2000). However, this was challenged by recent findings which suggested that there is only one type of PPV, and that Pex1-Pex6, are mainly involved in import of peroxisomal proteins (Knoops et al., 2015; Motley et al., 2015). Nevertheless, the idea that mature peroxisomes form by fusion of unique pre-peroxisomal structures is appealing, because it provides a mechanism for avoiding the mistargeting of peroxisomal enzymes to parent organelles (mitochondria and/or ER) (Kim, 2017).

Recently, ESCRT-III complex proteins were implicated in scission of PPVs from the ER into the cytosol (Figure 1A; Mast et al., 2018). However, ESCRT proteins are known for budding of vesicles away from the cytosol, for example in mutlivesicular body formation, or virus budding. Therefore, the role of ESCRT-III proteins needs further examination. Also, whether these proteins play a role in scission of all PPVs originating from the ER membrane or only a subset is not known.

We clearly do not completely understand PPV biogenesis from the ER membrane. There is no known mutant background that is completely devoid of PPVs, suggesting that there is redundancy among proteins involved in PPV formation. There are many outstanding questions in the field such as: how are PPVs generated? How do PMPs traffic from the ER or mitochondria to PPVs? Where do the RING and docking complex proteins assemble? We are beginning to understand some of these questions. We will discuss the recent advances in the exit sites for PPV biogenesis in Section “Links Between LD and Peroxisomes Biogenesis.”

## LD Biogenesis

Lipid droplets are ubiquitously present unique organelles that are enclosed in a phospholipid monolayer. Unlike other organelles, the LD core is made of neutral lipids such as TG and SE. Embedded in the phospholipid monolayer are more than 100 proteins, including enzymes that synthesize or degrade lipids for storage or energy, respectively (Ducharme and Bickel, 2008; Khor et al., 2013; Bersuker and Olzmann, 2018; Bersuker et al., 2018). In yeast, LDs remain permanently connected to the ER, whereas in mammalian cells at least some mature LDs are released from the ER (Jacquier et al., 2011; Olzmann and Carvalho, 2018). LD biogenesis begins with synthesis of neutrals lipids between the ER bilayer, by ER-associated neutral lipid synthesis enzymes (Jacquier et al., 2011; Kassan et al., 2013; Choudhary et al., 2015; Kimura et al., 2018). How these enzymes are sequestered at the sites of LD biogenesis is not known. Synthesis of neutral lipids leads to formation of a lens-like structure in the bilayer (Figure 1B; Choudhary et al., 2015). After an increase in the concentration of neutral lipids at these sites, they start demixing from the highly charged phospholipid bilayer giving rise to LDs (Choudhary et al., 2018). These LDs are covered with a phospholipid monolayer that eventually acquires several proteins that are required for maturation of LDs (Tan et al., 2014). However, no proteins other than the enzymes involved in synthesizing neutral lipids have been implicated in formation of nascent LDs, suggesting that LD biogenesis is a lipid-driven phenomenon. Indeed, it was demonstrated that the lipid composition of the ER at sites of LD formation regulates the formation of LDs (Zanghellini et al., 2010; Ben M’barek et al., 2017; Deslandes et al., 2017; Choudhary et al., 2018). It was shown that lipids such as lysophospholipids that generate positive intrinsic curvature favor LD budding, whereas lipids such as diacylgycerol (DAG) and phosphatidylethanolamine (PE) that induce negative intrinsic curvature disfavor budding. Lipids and proteins that affect membrane tension also influence the directionality of LD budding (Ben M’barek et al., 2017; Chorlay et al., 2017; Deslandes et al., 2017). There are protein families such as seipin, FIT and Pex30/MCTP2 that play critical roles in LD formation (Figure 1B; Fei et al., 2008; Choudhary et al., 2015, 2018; Grippa et al., 2015; Wang et al., 2016; Joshi et al., 2018). These proteins are at the sites of LD biogenesis and are required for efficient generation of LDs. Here, we discuss the current understanding of these proteins in LD biogenesis.

### FIT

The fat inducible transmembrane proteins (FITs) are conserved proteins that play a role in LD biogenesis. There are two homologs of FITs, FIT1, which is muscle specific, and FIT2, which is expressed in most tissues in mammals (Kadereit et al., 2007). In yeast, there are two FIT2 proteins, Scs3 and Yft2, whereas only one FIT protein is present in worms (Choudhary et al., 2015). Depletion of FITs leads to decreased LD biogenesis (Miranda et al., 2014). FIT proteins directly bind to TG (Kadereit et al., 2007). Depletion of FITs in yeast, mammals and worms results in LDs wrapped around with ER membrane suggesting a defect in budding of LDs (Choudhary et al., 2015). Later it was shown that in the absence of FITs, DAG levels in the ER increase, which might affect directionality of LD budding (Choudhary et al., 2018). In the same study, it was demonstrated that supplementation with exogenous lipids such as lyso-phosphatidylcholine and lyso-phosphatidic acid reversed the LD wrapping phenotype observed in the FIT mutants in yeast. Moreover, FIT protein, Yft2, along with DAG, transiently accumulates at sites of LD formation (Figure 1B). Thus, FIT proteins probably regulate DAG levels at the sites where LDs are formed. Maintaining the level of DAG in the ER membrane is vital as its accumulation could be toxic. Deletion of worm FIT and mouse FIT2 is lethal, supporting the importance of the cellular function of FIT proteins (Choudhary et al., 2015, 2018).

### Seipin

Seipin is a conserved integral ER membrane protein required for efficient LD biogenesis (Liu et al., 2016). The role of seipin in LD biogenesis was first identified in a screen performed in yeast to identify mutants with aberrant LD morphology. In two independent studies it was shown that in the absence of seipin, yeast cells exhibit multiple smaller, or fewer supersized LDs that are clustered together (Li et al., 2007; Fei et al., 2008). It was later shown that seipin is required for efficient incorporation of both proteins and lipids into the LDs (Salo et al., 2016). Seipin forms discrete foci in the ER membrane where nascent LDs are formed. Seipin along with Ldb16 in yeast is localized at ER-LD contact sites (Wang et al., 2014; Grippa et al., 2015). Seipin is highly mobile on the ER membrane until it encounters a nascent LD. Loss of seipin leads to smaller LDs that eventually fuse to form large and fewer LDs (Wang et al., 2016). Recent cryo-EM studies describe oligomeric structures of human (undecamers) and fly (dodecamers) seipin. Each monomer consists of a hydrophobic helix placed toward the ER bilayer and a β-sandwich domain which is structurally similar to lipid-binding proteins. It was shown that this domain binds to anionic phospholipids such as phosphatidic acid (Sui et al., 2018; Yan et al., 2018). Thus, seipin could regulate the local phospholipid levels at the site of LD budding.

### NVJ

Lipid droplet biogenesis occurs at specialized nuclear vacuolar junctions (NVJs) during nutrient stress. Mdm1, a molecular tether at the NVJ, and localizes at the site of LD formation. Overexpression of Mdm1 causes accumulation of LDs at the NVJ, supporting its role in LD biogenesis (Hariri et al., 2018). Other players in LD biogenesis at the NVJ include Ldo16 and Ldo45, which are overlapping genes with shared amino acid sequence. These proteins are required for accumulation of LDs at the NVJ during stationary growth phase. These proteins generate new LDs at NVJ under nutrient stress conditions to facilitate lipophagy, the breakdown of LDs through autophagy (Eisenberg-Bord et al., 2018; Teixeira et al., 2018). Whether spatial compartmentalization of LD biogenesis specific to nutrient stress occurs in higher eukaryotes remains to be investigated.

## Links Between LD and Peroxisomes Biogenesis

Several reports have suggested that LDs and peroxisomes are intimately associated (Schrader, 2001; Binns et al., 2006; Valm et al., 2017). Yeast cells grown in the presence of oleic acid exhibit peroxisomes that adhere stably with LDs by forming extended membrane processes called gnarls. These extensions were enriched in fatty acid beta oxidation enzymes, suggesting coupling of lipolysis within the LDs with peroxisomal enzymes (Binns et al., 2006). A recent report suggests that M1 spastin on the LDs physically tethers with ABCD1 on peroxisomal membranes. Moreover, M1 spastin recruits ESCRT III proteins to facilitate fatty acid trafficking from LDs to peroxisomes (Chang et al., 2019). Thus, there is a clear metabolic link between these organelles. In this section we discuss the shared machinery required for the *de novo* biogenesis and function of peroxisomes and LDs. Both organelles originate from the ER membrane (Joshi et al., 2017). Until recently, the sites in the ER membrane for formation of these organelles was not known. Also, it was unclear whether these sites form stochastically or are pre-determined.

### Pex30 Subdomains: Sites for Nascent LD and PPV Generation

Recent studies in yeast demonstrated that Pex30 localizes to discrete regions of the ER called ER subdomains. Pex30 is a resident ER protein, however, it localizes to peroxisomes or PPVs when cells are exposed to oleic acid or when Pex30 is overexpressed (Vizeacoumar et al., 2006; Joshi et al., 2016). Pex30 and Pex30-like proteins contain a reticulon homology domain (RHD), a transmembrane domain that is similar to reticulon proteins. Like reticulons, Pex30 proteins tubulate the ER membrane. However, unlike the reticulons, Pex30 and Pex30-like proteins are low abundance proteins localized to discrete regions in the ER. In cells devoid of peroxisomes, it was demonstrated using fluorescence and electron microscopy that newly expressed Pex14 is targeted to ER regions enriched with Pex30. Pex14 eventually leaves the ER membrane in a newly formed PPV (Joshi et al., 2016). This suggests that Pex30 subdomains are novel exit sites of nascent PPV formation (Figure 1A). Interestingly, these sites do not overlap with ER exit sites for COPII vesicles (Joshi et al., 2016). Whether other types of PPVs also form at these subdomains remains to be investigated. Deletion of Pex30 and the Pex30-like protein, Pex31, generates small clusters of PPVs closely associated with the ER membrane. The rate of formation of new peroxisomes is also decreased in cells devoid of Pex30 and Pex31 (Joshi et al., 2016). Thus, Pex30 and Pex31 are essential for efficient formation of peroxisomes.

**FIGURE 2:**
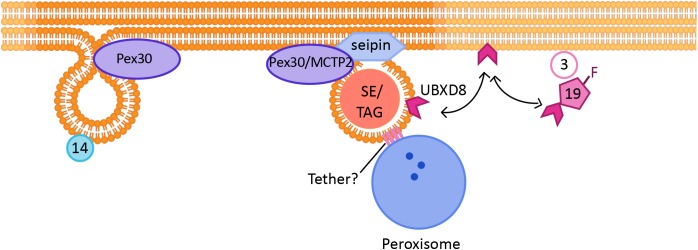
Link between peroxisome and LD biogenesis: The formation of new Pex14-containing PPVs and LDs occur at the same ER subdomains, which are positive for Pex30/MCTP2 proteins. Mature peroxisomes also physically associate with LDs. Tethers identified so far include ABCD1 on peroxisomes, which interacts with Spastin on LDs. Other tethers may also be involved in peroxisome-LD contacts. Pex19 is a cytosolic protein required for peroxisomal protein targeting, which when farnesylated targets a LD protein UBXD8 to LDs.

The number of Pex30 subdomains per cell is much greater than the number of PPVs per cell. Thus, Pex30 subdomains could have additional roles. Indeed, it was demonstrated that Pex30 subdomains are also the sites for nascent LD formation ((Figure 1B; Joshi et al., 2018). Furthermore, in yeast cells PPVs were associated with LDs at Pex30 subdomains, implying that PPV and LD biogenesis might occur at the same sites within the ER (Figure 2). It is possible that Pex30 also localizes to the site of new LD formation at the NVJ, as Pex30 is enriched at NVJ especially during stationary growth phase (Joshi et al., 2016). Pex30 subdomains exist even in the absence of LDs, suggesting that the sites at which LDs and PPVs form are stable, pre-determined, and not random. Pex30 has a functional homolog, multiple C2 domain containing transmembrane protein 2 (MCTP2), in higher eukaryotes, which also has an RHD (Joshi et al., 2018). Mammalian cells have two MCTP proteins, MCTP1 and MCTP2, whereas flies and worms have only one MCTP (Shin et al., 2005). Similar to Pex30, MCTP2 is also a low abundance protein that localizes to the sites of LD biogenesis (Figure 1B). MCTP2 subdomains also dynamically associate with the peroxisomal vesicles. However, MCTP2 does not localize to peroxisomes (Joshi et al., 2018).

It is possible that in addition to organelle biogenesis, these ER subdomains play roles in lipid trafficking and signaling. In the absence of Pex30, LDs are small and clustered and there is ER membrane proliferation. An independent study showed that loss of Pex31, a paralog of Pex30, also leads to smaller LDs (Lv et al., 2019). The rate of new LD formation was also significantly decreased in cells devoid of Pex30 (Joshi et al., 2018). It is fascinating that loss of Pex30 has similar morphological effects on both PPVs and LDs, suggesting that the mechanism involved in biogenesis of the organelles is shared (Joshi et al., 2016, 2018). Pex30 also colocalizes with several ER membrane proteins, including seipin, that are known markers for the sites of LD biogenesis. Deletion of seipin affected the distribution of Pex30 in the ER, as there was a significant decrease in the number of Pex30 punctae in the seipin mutant. Cells devoid of both Pex30 and seipin exhibited a severe growth defect, and highly clustered small and large LDs (Joshi et al., 2018; Wang et al., 2018). Seipin also affects peroxisome biogenesis, as deletion of seipin alone decreased the rate of formation of new peroxisomes (Wang et al., 2018). Thus, there is a clear link between LD biogenesis and peroxisome biogenesis.

### Shared Protein Machinery for Targeting of Peroxisomal and LD Proteins

Pex19, a well-established cytosolic chaperone protein, and Pex3, a PMP, are required for targeting of UBXD8, an LD protein, in the ER membrane of mammalian cells. UBXD8 is delivered to Pex3-enriched ER subdomains by farnesylated Pex19 ((Figure 2). UBXD8 was mistargeted to the peroxisome if Pex19 was not farnesylated, suggesting that the post-translational modification is vital to gain specificity of protein targeting (Schrul and Kopito, 2016). It is possible that enzymes that are required for LD maturation and growth follow the same route, as cells devoid of Pex3 or Pex19 exhibit smaller LDs (Wang et al., 2013). Another example of a link between peroxisomes and LDs comes from a recent study which demonstrated that in mammalian cells the peroxisomal protein fatty acyl CoA reductase 1 (Far1), is targeted to LDs upon increased triglyceride synthesis. Far1 exhibits two different topologies that differ in orientation of the short hydrophilic C-terminus toward the cytosol or lumen. Out of the two closely spaced hydrophobic domains, one is enough to target the protein to the LDs. Thus, Far1 exhibits dual topologies to peroxisomes and LDs dependent on cellular lipid metabolism (Exner et al., 2019).

## Conclusion and Future Perspectives

There are many open questions about the links between peroxisomes, LDs, and other organelles that originate from the ER. What is the common theme in the biogenesis of peroxisomes and LDs? Structurally, these organelles are very different. It is possible that Pex30 and seipin generate specialized ER subdomains for PPV and LD biogenesis. Seipin specifically binds to anionic phospholipids (Yan et al., 2018), whereas studies using sensor proteins such as the DAG-sensing PKD domain from mammalian cells and Dga1 protein demonstrated that Pex30 subdomains have enriched DAG and possibly phosphatidic acid, especially upon induction of LDs with oleic acid (Joshi et al., 2018). In addition, the LD defect in cells devoid of seipin and Pex30 was rescued by manipulating ER phospholipid composition (Wang et al., 2018). Thus, the generation of these organelles could be influenced by lipids. Pex30 could induce curvature with its RHD, which might be conducive for the synthesis of certain lipids to facilitate the generation of LDs and PPVs. It is also possible that the biogenesis of these organelles from a common subdomain allows their formation to be coordinated in response to stimuli that require the proliferation of both, such as a lipid challenge. It has been demonstrated that Pex30 subdomains do not overlap with ER exit sites. However, it remains to be determined whether these domains play a role in the biogenesis of other organelles that originate from the ER, such as autophagosomes, which can also be induced in response to excess lipids (Singh et al., 2009). There are hints that LD and autophagosome biogenesis may be related: both autophagosomes and LDs have been observed cradled within “cups” of ER membrane, and LC3 and Atg2, which are involved in autophagosome biogenesis, also localize to LDs (Robenek et al., 2006; Hayashi-Nishino et al., 2009; Shibata et al., 2009, 2010; Velikkakath et al., 2012). These observations suggest that autophagosome and LD biogenesis may share some common machinery, but whether Pex30 ER subdomains are involved in autophagosome biogenesis is currently unknown.

Finally, the ER subdomains involved in organelle biogenesis may also be membrane contact sites through which peroxisomes and LDs communicate with the ER after their biogenesis. In this case they could play roles in organelle remodeling, or in the trafficking of proteins and/or lipids between the ER, peroxisomes, and LDs. Peroxisomes in mammalian cells are tethered to the ER via ACBD5-VAP proteins. This tether is essential for peroxisomes to receive lipids from the ER required for peroxisomal membrane expansion (Costello et al., 2017; Hua et al., 2017). Several tethers have been proposed for ER-LD contact sites. These include interactions between NRZ-SNARE on the ER and Rab18 on LDs in mammalian cells, and between the lipid synthesis enzymes FATP1 on the ER and DGAT2 on LDs in worms (Xu et al., 2012, 2018). Whether Pex30 ER subdomains colocalize with any of these tethers, or whether they form distinct membrane contact sites, is currently unknown. The precise protein composition of these ER-LD-peroxisome membrane contact sites, their physiological function, and regulation all remain to be determined. The recent development of new biochemical and microscopic tools to study membrane microdomains and membrane contact sites is sure to open up many new areas of research in this exciting field.

## Author Contributions

AJ and SC conceived and wrote the manuscript.

## Conflict of Interest Statement

The authors declare that the research was conducted in the absence of any commercial or financial relationships that could be construed as a potential conflict of interest.
